# Some students are more equal: Performance in Author Recognition Test and Title Recognition Test modulated by print exposure and academic background

**DOI:** 10.3758/s13428-023-02330-y

**Published:** 2024-01-09

**Authors:** Marion Hug, Julian Jarosch, Christiane Eichenauer, Selina Pennella, Franziska Kretzschmar, Pascal Nicklas

**Affiliations:** 1 Department of Neurology, University Hospital Frankfurt, Frankfurt am Main, Germany; 2 Institute of Anatomy, Neuroimaging Center (NIC), University Medical Center Campus, Building 308 C, Langenbeckstrasse 1, 55131 Mainz, Germany; 3 Academy of Sciences and Literature, Mainz, Germany; 4 Department of English and Linguistics, Johannes Gutenberg University Mainz, Mainz, Germany; 5 Gutenberg Institute for World Literature and Written Media, Johannes Gutenberg University Mainz, Mainz, Germany; 6 Department of Grammar, Leibniz-Institute for the German Language, Mannheim, Germany; 7 Department of Book and Reading Studies at the Gutenberg Institute for World Literature and Written Media, Johannes Gutenberg University Mainz, Mainz, Germany

**Keywords:** Author recognition test, Title recognition test, Print exposure, Reading, Individual differences

## Abstract

Reading is a key skill for university students. The Author Recognition Test (ART) and the Title Recognition Test (TRT) have both been used for decades to measure print exposure which correlates with reading and other linguistic skills. Given the available evidence for interindividual differences in reading skills, this study addresses three open issues. First, to what extent do ART and TRT scores correlate with individual differences regarding students’ study programs? Second, how do these results correlate with the self-reported time spent reading different types of text genres (e.g., fiction, nonfiction) per week? And third, this study compares ART and TRT to one another. We tested students from six study programs in the humanities and (medical) sciences which vary in the amount and kind of reading material required for study success. We found that students perform significantly differently in the ART and the TRT depending on their field of study. Students in a study program focusing on fiction and literature perform best overall. We also replicated the well-known effect of age on ART and TRT scores: older students have better scores. We did not find reliable effects of reading time on test performance, whereas individual creative writing habits did positively predict ART/TRT test results. These results raise a number of important questions regarding the ART/TRT in general and regarding interindividual differences in personal reading and writing habits and the change in reading habits in times of media convergence.

## Introduction

Reading is a key skill in cultures with a written language. As a cultural skill, it changes the brain’s neural networks, (Dehaene, 2009), improves theory of mind (Dodell-Feder & Tamir, 2018) and social behavior (Mar, 2018; Oatley, 2016), and correlates with emotional (Billington, 2016, 2019; Schwering et al., 2021) and cognitive (Wolf, 2007, 2018) skills. Like any other skill, reading requires considerable practice. The more you read, the better you become at reading and at many other activities as well (Cipielewski & Stanovich, 1992). Therefore, the acquisition of reading (and literacy) skills in cultures with a written language is a major reason for inter- and intraindividual differences in humans and their use of language.

In reading research, print exposure, defined as the “amount of text” (Acheson et al., 2008: 278) read by an individual reader, is used to estimate participants’ exposure to written language across their lifespan. It is of interest as it allows us to predict specific linguistic skills and can give some idea of the level of reading expertise (Allen et al., 1992; Acheson et al., 2008; Mol & Bus, 2011). It has been found to correlate with orthographic and phonological skills (Chateau & Jared, 2000), reading comprehension (Calloway et al., 2022; Cipielewski & Stanovich, 1992; Vermeiren et al., 2022), nonword naming (McBride-Chang et al., 1993), vocabulary size (Frijters et al., 2000), reading and verbal ability in college students (Acheson et al., 2008), and generally with the language and reading skills of children, adolescents, and young adults (Mol & Bus, 2011). Here, we focus on the question as to what extent the most prominent measures of print exposure, the Author Recognition Test (ART) and Title Recognition Test (TRT), interact with the characteristics of participants and test items.

The measurement of print exposure retrospectively or in terms of lifetime reading has proven difficult. Self-report measures such as questionnaires or activity diaries are problematic because they are susceptible to social desirability in participants’ answering strategies and, hence, a lack of reliability of participants’ answers (Paulhus, 2002). Interference from social desirability factors may therefore make it more difficult to generalize individual reading behavior retrospectively (McCarron & Kuperman, 2021). About three decades ago, the ART was developed to assess print exposure in individuals without the problems stemming from self-report measures (see the discussion in Stanovich & West, 1989). Ever since it has been the standard test, complemented by the TRT or the Magazine Recognition Test (MRT) (Cunningham & Stanovich, 1990; Kim & Krashen, 1998) for some other purposes (Acheson et al., 2008; Cunningham & Stanovich, 1990; Grolig et al., 2020; Stanovich & West, 1989). The basic procedure of the ART appears straightforward: Participants are presented with a set of real and fake author names and are asked to identify the real authors. The key result is the successful distinction between real and fake authors. In the TRT/MRT, author names are replaced by titles.

Despite the ubiquitous use of ART/TRT measures in experimental reading research (Wimmer & Ferguson, 2022), a number of mediating factors have received insufficient attention in the literature, thereby challenging a straightforward interpretation of the ART results. A pertinent example in this regard is age. A recent study by Grolig and colleagues revealed that age plays an important role in test performance, but evidence for its precise impact is inconclusive (Grolig et al., 2020). The authors argued that one reason may be that participants for the ART are usually recruited from university students, thus limiting experimental sensitivity for age effects on ART results. Their findings indeed show that sampling a wider age range supports the exploration of the effects of age on ART results. Grolig and colleagues recruited university students and visitors to the Frankfurt Book Fair, resulting in a sample with an extended age range of 13 to 77 years. The authors found that age specifically influenced test performance for “classic” authors whose mean publication year was earlier: the older the readers were—i.e., the more reading experience they had—the better their results for “classic” authors. For recent authors, however, this increase was only seen between adolescence and middle age. It remains to be seen how age interacts with other participant characteristics that may influence what and how much someone may have read throughout life.

One such participant characteristic is the academic background, which has not been taken into account yet for ART (or TRT) evaluation. Often, the ART is performed with a convenience sample consisting of psychology students; however, the academic program in which students are enrolled should make a difference in their ART performance. This is because course requirements directly affect the amount of reading and kinds of text. For instance, in literary studies, the reading of novels, poetry, drama, diaries, and essays is a requirement for any student. Students in other programs might have to read and memorize textbooks and science papers for their studies, while they read novels in their spare time for relaxation or out of interest. As a consequence, students of literature can be expected to spend relatively more time reading fiction and, importantly, may read more works by authors whose works are the test material for the ART compared with, for example, students of psychology or medicine. Also, the preference for reading materials (novels, newspapers, journals, etc.) in the student population has been looked at (Alsaeedi et al., 2021). It is not unreasonable to expect that it makes a difference what you read: if you prefer popular magazines or hermeneutically difficult academic texts, this will have an effect on your reading skills and make a difference in your literacy or analytical skills. Therefore, the performance in the ART (or TRT) might vary depending on the academic background and reading preferences of the individual tested.

Without further scrutiny, convenience sampling of university students for ART (or TRT) measurements may therefore result in a heterogeneous group of participants whose demographic or habitual characteristics are not well controlled for and who may in turn generate experimental results that do not generalize to other populations (Henrich et al., 2010; Rad et al., 2018) because of sampling bias due to educational, cultural, or socioeconomic backgrounds.

Assessing the impact of academic fields on test performance also addresses the open question of whether differences in students’ mnemonic strategies for remembering information from written texts differ as a function of the kinds of text to be read—which basically translates into a direct comparison of ART and TRT measurements. For the nonfiction genre including university textbooks, for instance, titles may better cue the contents of a book than its author. This conjecture is motivated by research on print exposure in children that has assumed that children remember the titles of books rather than the authors’ names, and correspondingly, found that the TRT is a reliable predictor of orthographic or phonological processes in word recognition (Cunningham & Stanovich, 1990). Thus, the reliability of the TRT might be different from that of the ART depending on whether the study program student participants are enrolled in has a focus on fiction (e.g., literary studies) or nonfiction genres (e.g., psychology).

Alongside participant characteristics, a second major point of contention is the selection of real authors or titles (i.e., test items). They can be selected based on criteria such as sales figures, canonicity, or genre. In their seminal work on the ART, Stanovich and West (1989) did not opt for canonical authors in selecting the real author names, but looked at recent sales figures. This has become the standard in later tests developed by other researchers (Acheson et al., 2008; Chateau & Jared, 2000; Chen & Fang, 2015; Grolig et al., 2020; Masterson & Hayes, 2007). Stanovich and West argued against selecting “highbrow” authors, expecting them to be known only by the “most highly educated or academically inclined readers” (Stanovich & West, 1989, p. 408). Hence, their list reflected mechanisms of the book market and choices made by actual book buyers and readers. The reasoning behind selection by sales figures was that exposure to printed matter would result in coming across these names.

Yet, it was not necessary to have actually read books by the selected authors to score well in the ART, because knowledge of authors’ names could also have been obtained, for example, by having read *about* the authors and their works (in book reviews, etc.). The problem of knowing an author’s name without having read any of their books is amplified through the current practices of media consumption, which increases information flow. Stanovich and West developed the ART in a completely different cultural and educational world compared with the culture of convergent media in the third decade of the twenty-first century. The ART, covering familiarity with a specific kind of written culture, i.e., fiction and information in book form, does not cover print exposure in other forms such as textbooks or online reading, which also differ in terms of the processing of what is read depending on different text genres or forms of presentation (Baron & Mangen, 2021; Mangen et al., 2019).

Technological advancements have both increased and altered the ways we read (and write) nowadays. In contrast to the book reading that Stanovich and West had in mind, reading nowadays does not only take place when one sits down with a book or scientific paper. Rather, it is performed mostly on the computer and even more so on the cell phone screen (Stiglic & Viner, 2019). It often includes interactive and multimedia content with concurrent spoken and written language and may yield different instances of “deep” and “superficial” reading (Wolf, 2018; Wolf & Barzillai, 2009). Also, there are effects of specific reading devices (Kretzschmar et al., 2013; Mangen et al., 2019; Mangen & van der Weel, 2016). Similarly, current media practice may put more emphasis on individual literary products rather than their producers, again pointing to possible differences between ART and TRT. To date, there is no method to reliably take these forms of print exposure into account, as screen time does not account for reading alone (Schutte & Malouff, 2004). Still, data from self-report measures about *what* someone reads or about habitual ways of reading and writing in media other than the book can be informative.

In summary, the current study investigates performance in the ART and TRT with regard to three related research questions. First, we recruited student participants from six different fields of study: comparative literature, English studies, German studies, linguistics, medicine, and psychology. This allowed us to address whether print exposure measured in the ART/TRT is influenced by the academic background of the participants.

If print exposure is tied to a specific genre, we would expect a dissociation between the humanities and sciences. As the ART covers more fiction than nonfiction authors due to its selection criteria, humanities students should perform better because they read more fiction as part of their study requirements than do students in psychology or medicine.

There may also be qualitative differences between academic fields within the humanities. Students of comparative literature read texts from diverse written cultures and translations from various languages, but also texts written in their native German. Students of German and English studies, by contrast, selectively read fiction from either German- or English-speaking authors. Linguistics is concerned with language but not literature. Also, the courses are organized in very different ways concerning the focus on literature or culture. English and German studies focus on cultural contexts or explicitly do cultural studies, while comparative literature might include media studies but does not include cultural studies. Thus, comparative literature is different from German or English studies in its much clearer focus on fiction, and therefore a presumably higher level of print exposure to fiction. This predicts that students of comparative literature may perform better in terms of recognizing fictional authors than students from other academic disciplines, given that fiction is a crucial aspect in the selection of authors or titles in the ART and TRT.

Second, we developed a TRT which has been constructed analogously to our ART with regard to the composition of genres and the time of the writing of the books. The results from the two tests, thus, become closely comparable, so that we can pursue the question of whether recognition of content (mnemonically associated with the title) dominates over meta-literary information (the author name) or vice versa. This allows us to test whether academic background influences the way participants memorize information about print material.

Third, we included a self-report questionnaire on reading and writing habits in order to compare the ART and TRT scores with qualitatively different, independent measures. If only the “amount of text” (Acheson et al. 2008, p. 278) that readers read matters for test scores, students may not differ based on their academic background, as students from all study programs read comparable amounts of fiction or nonfiction text as part of their study requirements. However, individual practices of media consumption may then become a predictor of test performance. We therefore asked participants in a questionnaire to indicate what genre(s) they read and what they write if they write creatively themselves; we then correlated their answers with ART/TRT test scores.

## Method

### Participants

The overall size of our participant sample is in accord with the sizes reported in previous exemplary studies ranging from 188 (Stanovich and West 1989: a single study) to 1012 (Moore and Gordon 2015: collected in 23 different experiments). Most recent studies report about 200 participants: Grolig and colleagues (Grolig et al., 2020) analyzed a sample of 231. Where Vermeiren and colleagues (Vermeiren et al., 2022) analyzed the ART in their series of experiments, sample sizes ranged from 182 (their study 5) to 195 or 196 (their studies 1 and 3, respectively). Calloway and colleagues (Calloway et al., 2022) report 187 participants in the second of their studies that uses the ART.

In our study, 410 participants from three German universities (University of Cologne, Goethe University of Frankfurt, and Johannes Gutenberg University of Mainz including the University Medical Center of the University of Mainz) participated after giving informed consent. Prior to their participation, they were informed about the purpose of the study. Participants were free to withdraw from the study at any time. In accordance with the rules of the German Research Foundation (DFG), the study was not accompanied by an ethics vote. Psycholinguistic experiments involving healthy adult participants and behavioral noninvasive methods for data collection are exempt from an ethics vote as long as they pose no risk or physical/emotional burden to participants (https://www.dfg.de/foerderung/faq/geistes_sozialwissenschaften/).

**Fig. 1:**
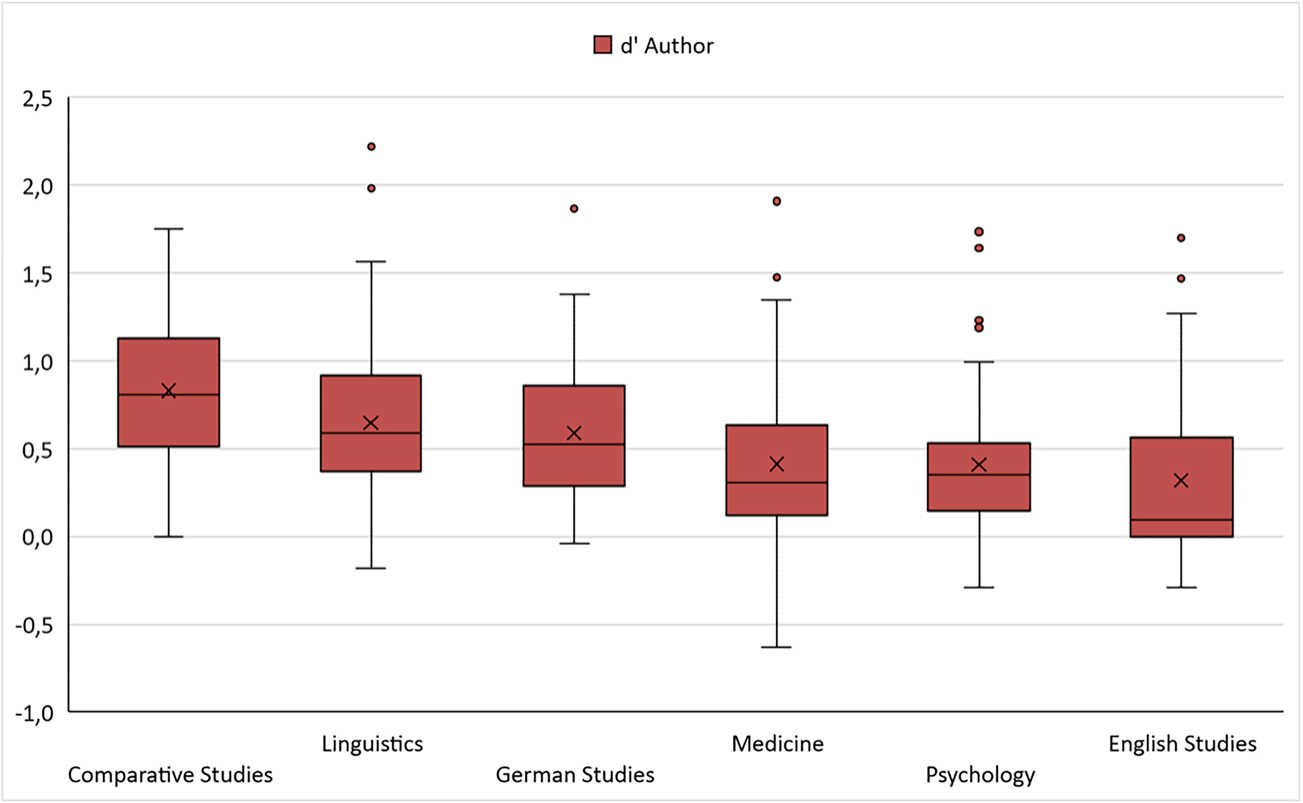
Boxplot d′ per field of study for ART

**Fig. 2:**
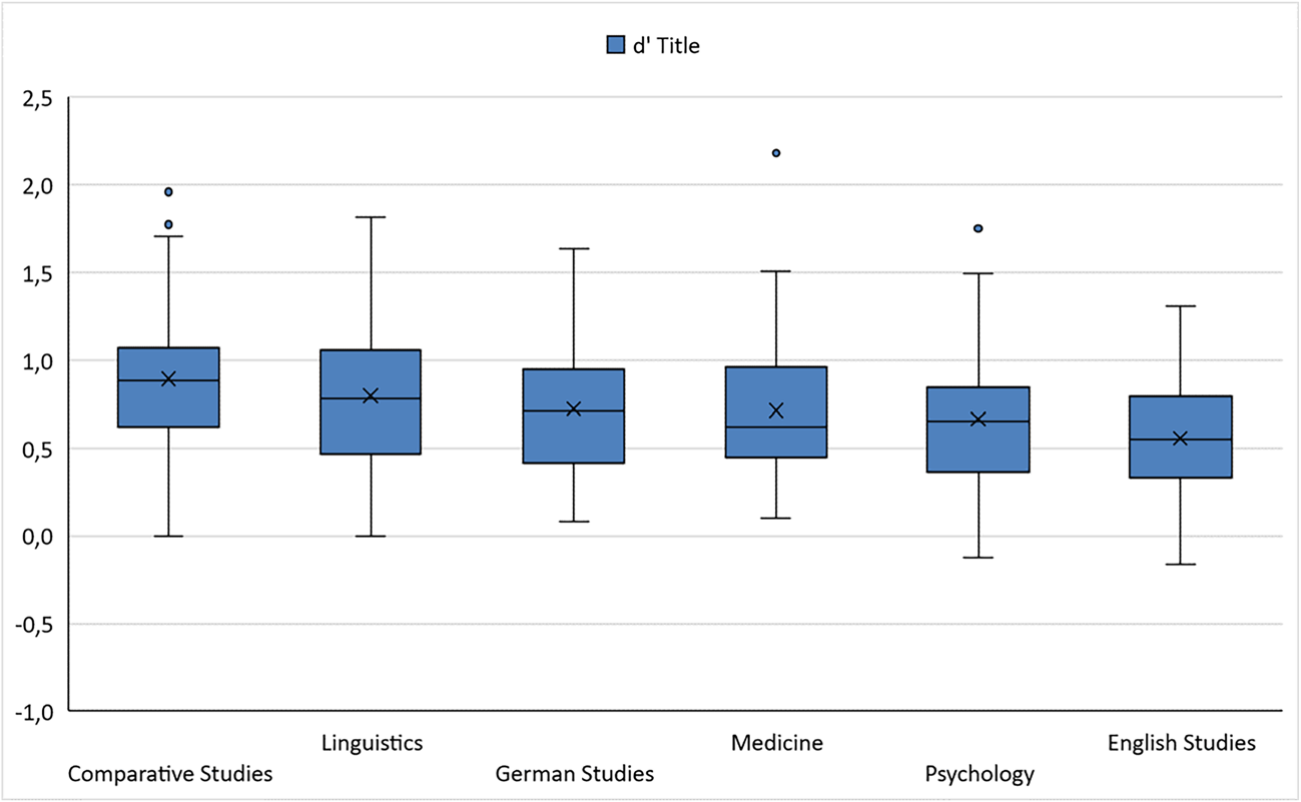
Boxplot d’ per field of study for TRT

Out of the 410 questionnaires, 107 were excluded for the following four reasons: (a) if the answering behavior was aberrant (mostly questionnaires where all the questions were answered in the same way, such as all answers were “yes”) (50 questionnaires = 12.2%); (b) if more than 10% of the answers in either the ART or TRT were missing (9 questionnaires = 2.2%); (c) if the students did not study one of the aforementioned six academic subjects (25 students = 6.1%); (d) if the students were not native speakers of German or did not indicate that German was their dominant language (23 participants = 5.6%). Criterion (d) is based on a previous finding that ART performance drops significantly for non-native speakers (McCarron & Kuperman, 2021). This resulted in a sample of 303 participants for further analysis (see the supplementary materials: https://osf.io/s6j2e/?view_only=7e8f0f874570449eb7ce0865f6d9e38b), with 45 to 57 participants per field of study (see Table 1). Note that “Comparative literature” equals “Comparative studies” in text and figures, particularly in Figs. 1, 2 and in Table 4 below.

**Table 1:** Number of participants per field of study

Field of study	Number of participants
English studies	57
Psychology	55
Medicine	52
German studies	47
Comparative literature	47
Linguistics	45
Total	303

Of these participants, 236 students described themselves as “female” (77.9%), 65 students described themselves as “male” (21.5%), and two students described themselves as “diverse” (0.7%). The mean age was 30 years (range 18–48, standard deviation [*SD*] = 3.6).

### Materials

Three questionnaires were developed and given to each participant. One asked for demographic data and reading and writing habits. The other two were German versions of the ART and of the TRT.

### Questionnaire on demographic data and reading and writing habits

The questionnaire has two vital functions. First, it identifies the academic subject in which the participant is enrolled. Additionally, it asks for demographic data: age, handedness, sex, native language, educational status. Second, it provides a self-assessed identification of reading habits and print exposure. Participants had to indicate the number of hours per week they spent reading each of the following genres: nonfiction, novels, poetry, biographies, newspapers, journals, scientific articles, emails, online texts, and an open-ended category “other.” Note that for later analysis, these genres were grouped into fiction (novels, poetry, biographies), nonfiction (nonfiction, newspapers, journals, scientific articles), and online reading (web texts, emails). The scale had a numeric range from 0 to 7, and its maximum endpoint was labeled with “more.” The questionnaire also asks whether the participants write themselves (yes/no question with further subdivision for yes answers: poetry, stories, and other) and what their favorite author or genre is (with an open-ended format). See the example questionnaire in the supplementary materials: https://osf.io/s6j2e/?view_only=7e8f0f874570449eb7ce0865f6d9e38b.

### Author Recognition Test

The ART was developed by authors CE, JJ, and FK. Following a previous version of the ART for U.S. English (Acheson et al. 2008), the items for the author recognition test consisted of 65 real author names and 65 foils. The selection of authors for our ART did not make use of any previous ART for German speakers. We used two criteria for selection: sales figures and school curricula. Sales figures reflect current popularity, while school curricula refer to canonical values and cultural–educational knowledge of a more established kind. While Stanovich and West (1989) expressly excluded school curricula in order to test free reading habits, we tried to establish the possible significance of curricular authors as compared with sales figures.

Possible authors for the ART were identified according to three criteria: (1) they belonged to the list of the top 20 bestsellers between 2005 and 2015 for fiction and nonfiction writing, (2) they were among the top 50 bestsellers listed for 2015, or (3) they received well-known national or international prizes for their work (e.g., the Nobel Prize), which also ensures significant sales. For this process we used the most popular bestseller lists in Germany (Spiegel Bestseller lists; https://www.buchreport.de/spiegel-bestseller/) which come out weekly and are averaged on a yearly basis by buchreport.de. These lists incorporate sales figures for various book types (e.g., hardcover, paperback, audiobooks) and points of sale (e.g., online and local bookshops or bookshops at train stations).

Additionally, we selected a small number of authors whose work is part of a curriculum in secondary schools in at least one of the German Federal States. We were careful to not select authors who are deeply ingrained in Germany’s general cultural knowledge, i.e., authors whose names anyone would know without having ever read one of their works, e.g., Johann Wolfgang von Goethe or Friedrich Schiller.

Table 2 shows the distribution of real and foil names according to the variables gender, original language of publication, text genre, and whether the author’s work is part of a curriculum in secondary schools in at least one of the German federal states. Since the majority of authors are selected based on socioeconomic criteria (sales figures, reputation), which tend to bias towards a certain gender and genre distribution, we chose to not correct for these biases in our list of authors in order to maintain a modest level of external validity. Therefore, more than two thirds of the real authors are male. The ratio between male and female author names is slightly greater even for the foils. Collapsed across real and foil authors, there are 65.38% male names and 33.85% female names, which is similar to the gender distribution in earlier ART versions (Stanovich & West, 1989). One foil is not associated with a binary gender because it contains initials only (“R.M. Moya”) to match with one of the actual author names (the pseudonym “E.L. James”).

**Table 2:** Item characteristics

	Real authors (*N* = 65)	Foils (*N* = 65)
Gender		
‐ Male	49 (75.38%)	36 (55.38%)
‐ Female	16 (24.62%)	28 (43.08%)
‐ NA	0	1 (1.54%)
Original publication language		
‐ German	45 (69.23%)	40 (61.54%)
‐ Other	20 (30.77%)	25 (38.46%)
Genre		
‐ Fiction	41	NA
‐ Nonfiction	24	
Part of school curriculum in Germany	19 (29.23%)	NA

We coded two broad genres: fiction (including, e.g., novels, short stories or other forms of prose, and poetry), and nonfiction (including books and articles). Authors were grouped according to the genre of their book scoring highest in sales figures. This grouping thus purposefully ignored the fact that authors publish in different genres. Text genres are unevenly distributed; the majority of authors are fiction writers (see Table 2).

Finally, 19 out of the 65 authors were part of a teaching curriculum at secondary schools in at least one of the German federal states. We had information about 14 curricula (out of the 16 German states, which each have their own curriculum) to check which of our authors were taught at schools. (The remaining two German states did not provide specific reading lists for their curriculum.) Table 3 gives the frequency of authors in the curriculum, ranging from Nobel Prize winner Günter Grass (12 out of 14 curricula) to four authors present only in one curriculum.

**Table 3:** Authors taught at secondary schools in Germany (canonical authors)

Author	School curricula (no. of German states; *N* = 14)
Grass, Günter	12
Bachmann, Ingeborg	10
Remarque, Erich Maria	10
Böll, Heinrich	9
Hesse, Hermann	9
Döblin, Alfred	8
Süskind, Patrick	8
Rilke, Rainer Maria	7
Kehlmann, Daniel	5
Schlink, Bernhard	5
Tucholsky, Kurt	5
Saint-Exupéry, Antoine de	4
Müller, Herta	3
Ende, Michael	2
Ringelnatz, Joachim	2
Houellebecq, Michel	1
Modiano, Patrick	1
Rowling, Joanne K.	1
Schmid, Wilhelm	1

In our sample of real authors, the majority publish their work in German and only about one third originally publish in English or French (cf. Table 2). These works are available in German translations. As this criterion, of course, does not apply to the foils, we decided to signal their original publication language in terms of name composition. For the “German” foils, we used common first names with typical spellings and unambiguous gender marking. The foil surnames were all existing family names or, in one case, street names. The “international” foil names included first and last names of English, Swedish, and French origin. The ratio between “German” and “international” foils was comparable to that for real authors publishing in German versus another language. We checked foil names on the web to prevent cases where we inadvertently picked a real author name.

Traditionally, participants are asked to put a check mark (Stanovich & West, 1989) next to the author name they recognize or to circle the name (Moore & Gordon, 2015). As an alternative to the traditional method of the ART, we chose the two-alternative forced-choice (2afc) method to circumvent the problems of the response bias. The 2afc method forces participants to make up their minds by having to give an answer, be it positive or negative. To facilitate choosing between “yes” and “no,” we offered four choices. After each name or foil, there were four possible answers which could be ticked: (1) *this is certainly an author*, (2) *this is presumably an author*, (3) *this is presumably not an author*, (4) *this is certainly not an author*. Each of the answers had a color-coded box: *certain* (green), *presumably* (pale green), *presumably not* (pale red), and *certainly not* (red). Participants were asked to make up their minds about the correct answer and not to guess, but to always check one of the four boxes.

### Title Recognition Test

The TRT was developed based on the ART. The bestselling book title of all real authors was identified using Amazon sales figures without any concern for the genre of the book, resulting in 24 nonfiction and 41 fiction titles. Therefore, the ratio of fiction/nonfiction is not balanced by design, but originates from the bestselling book selection. For the foils, titles were created by co-author SP and discussed and amended by co-authors MH and PN. Twenty-four foils corresponding to real nonfiction books were worded so that they stylistically sound like nonfiction. Forty-one foils were worded to sound stylistically like typical fiction titles. As in the ART, we ensured that the title foils really do not exist. The order of titles in the TRT questionnaire corresponds to the order of author names and foils in the ART questionnaire. In the TRT, the same four different answers were possible as in the ART, the same color coding was applied, and the scoring occurred according to the same principles.

### Procedure and analysis

For the ART and TRT, real names/titles and foils were presented in random order. The test was administered in a paper-and-pencil format. In order to minimize sequence effects, two versions were used: version A presented the ART before the TRT, version B presented the TRT before the ART. In both versions, participants first answered the questionnaire on demographic data and reading habits (see the example questionnaire in the supplementary materials: https://osf.io/s6j2e/?view_only=7e8f0f874570449eb7ce0865f6d9e38b). Participants were randomly assigned to one of the two versions.

The data were obtained in three different contexts: One set of data was collected prior to psycholinguistic experiments conducted in the linguistic Neuro-Lab at the University of Mainz and the xLinC lab at the University of Cologne. The majority of data were collected at the end of seminars by the person teaching the seminar or by someone joining the seminar only for the testing. These end-of-seminar testing sessions took place in Mainz and Frankfurt. The first questionnaires were collected in May 2016. The last questionnaires were completed in November 2019. The application of all three tests took about 20 minutes including instruction.

For ART/TRT scoring, we calculated the sensitivity index d′ to investigate the distinction between real names and foils in the case of ART and between real titles and foils in the case of TRT. This index is the standard method of scoring in the signal detection paradigm (Wickens, 2001). It is estimated on the basis of the hit rate (the relative number of authors correctly identified) and false alarm rate (the relative number of foils incorrectly selected), as given in d′ = Z (hit rate) – Z (false alarm). From this, it follows that d′ = 0 represents perfect guessing, which results in the exact same amount of guessing correctly and falsely. Participants who are knowledgeable will have a positive value which increases with each correct answer. Correct answers reflect a correctly identified name or correctly rejected foil. In order to follow the original 2afc methodology, we coded both answers “certainly” and “presumably” as “yes,” while “presumably not” and “certainly not” were coded as “no.”

The data were analyzed in SPSS (version 27). We ran two types of models for ART and TRT scores. First, d′ values per participant were analyzed using a linear regression, with the fixed effects field of study (six levels: comparative literature, German studies, English studies, linguistics, psychology, medicine), gender (male vs. female vs. diverse), handedness (left-handed vs. right-handed), age, educational status (“Volksschule” [grade school, 8 years of schooling], “Hauptschule” [secondary modern school, 9–10 years], “mittlere Reife” [General Certificate of Secondary Education (GCSE), 10 years], “Abitur” [high school diploma, 12–13 years], BA degree, MA degree, no educational degree), reading habits (overall reading time [hours/week] for fiction, nonfiction and online texts), and creative writing (yes vs. no).

We used treatment coding for the (categorical) fixed effects; the reference level was the factor level with the highest number of responses: field of study (reference level: English studies), gender (reference level: female), handedness (reference level: right-handed), educational status (reference level: “Abitur” high school diploma), creative writing (reference level: no). We used a stepwise procedure to test for the inclusion of fixed effects in the model. The selection criterion for retaining a predictor was *p* ≤ .050, and the criterion for omitting a predictor was *p* ≥ .100. Therefore, the model outputs only include significant predictors.

Second, in order to determine which items of the ART or TRT could be used to predict the overall ability of participants to identify real authors or titles as measured by d′, we ran further linear regression models based on the ratings given per item. Note that, here, the type of hierarchical coding makes use of the full rating scale, from 1 = *this is certainly an author/title* to 4 = *this is certainly not an author/title*. This results in a high numerical value being “false” for real authors/titles, possibly leading to a reduction in d′. Conversely, for the fake authors/titles, a high numerical value is the correct answer and should accordingly lead to an increase in d′. Accordingly, when item-based answers from the questionnaire are used as a predictor of d′, a negative beta weight would be expected for real authors/titles and a positive beta weight for foils.

## Results

### Reliability scores

We checked the reliability of both tests. The ART had a reliability score (Cronbach’s alpha) of 0.957 (130 items), and the TRT had a score of 0.943. Thus, both tests show a very high level of internal consistency.

### Descriptives: ART/TRT scores and questionnaire on demographic data and reading and writing habits

Overall, the mean sensitivity index (d′) in the ART was 0.521, with a standard deviation of 0.476 (range: −0.631 to 2.218). In the TRT, the mean d′ was 0.717 (SD: 0.407, range: −0.163 to 2.179). Figures 1 and 2 show box-and-whiskers plots with median d′ values for both ART and TRT, split by field of study (see also Table 4). Descriptively, ART and TRT scores were best for students of comparative literature, while students of English studies performed worst in both tests. TRT scores were higher than ART scores overall. For the self-report measures, Table 4 shows that the total reading time per week was 18.1 hours across all participants. Most of this time was spent reading online texts and nonfiction. A total of 104 out of 303 participants (34.3%) stated that they engaged in creative writing. There was great variety concerning fields of study: whereas 66.0% of students of comparative literature wrote fiction, only 9.6% of medical students wrote fiction.

**Table 4:** ART and TRT scores per academic field of study, mean reading time, and percentage of participants engaged in creative writing

Course of study	Total	Comparative studies	Linguistics	German studies	Medicine	Psychology	English studies
d’ Author							
- Maximum	2.218	1.752	2.218	1.867	1.907	1.734	1.701
- Third quartile	0.812	1.130	0.917	0.856	0.636	0.530	0.560
- Mean	0.521	0.830	0.647	0.588	0.414	0.410	0.319
- Median	0.433	0.807	0.589	0.522	0.306	0.349	0.092
- First quartile	0.157	0.514	0.369	0.288	0.121	0.147	0.000
- Minimum	−0.631	0.000	−0.179	−0.039	−0.631	−0.293	−0.290
d’ Title							
- Maximum	2.179	1.959	1.308	1.634	1.959	2.179	1.752
- Third quartile	0.954	1.073	0.794	0.950	1.055	0.962	0.846
- Mean	0.717	0.894	0.556	0.722	0.796	0.715	0.667
- Median	0.677	0.885	0.548	0.712	0.784	0.621	0.653
- First quartile	0.412	0.622	0.331	0.415	0.467	0.449	0.366
- Minimum	−0.163	0.000	−0.163	0.083	0.000	0.098	−0.122
Reading (h/week)							
- Total, mean (%)	18.1 (100)	21.8 (100)	19.9 (100)	19.7 (100)	15.4 (100)	15.3 (100)	17.5 (100)
- Online, mean (%)	6.3 (35.4)	6.6 (30.8)	7.4 (36.9)	7.0 (36.0)	4.5 (30.0)	5.7 (37.6)	6.8 (40.2)
- Fiction, mean (%)	3.6 (19.1)	5.9 (28.8)	3.3 (16.6)	4.2 (22.0)	1.4 (7.6)	2.9 (17.5)	4.1 (22.5)
- Nonfiction, mean (%)	8.2 (45.5)	9.3 (40.4)	9.2 (46.5)	8.4 (42.0)	9.5 (62.4)	6.8 (44.9)	6.6 (37.4)
Creative writing							
- Yes, n (%)	104 (34.3)	31 (66.0)	16 (35.6)	23 (48.9)	5 (9.6)	12 (21.8)	17 (29.8)

When the four categories on the rating scale are displayed separately, we see that on average, participants accept real authors with high certainty in about 17.5% of all cases, whereas they accept them with less certainty in 12.8% of all cases (cf. Table 5). For real titles, we find the same relative pattern, albeit with higher values (as expected since the TRT performance was better overall). Interestingly, for foils, participants were in general less confident in their responses, as indicated in the higher numbers of “presumably” responses.

**Table 5:** Distribution of responses for real and fake items in both tests (upper panel) and choice of endpoint categories

Chosen score (%, mean)	1	2	3	4
ART real author (1/2 = hit, 3/4 = miss)	17.5	12.8	16.3	3.3
ART fake author (1/2 = false alarm, 3/4 = corr. rejection)	3.2	18.5	23.5	4.7
TRT real title (1/2 = hit, 3/4 = miss)	21.7	14.1	11.5	2.5
TRT fake title (1/2 = false alarm, 3/4 = corr. rejection)	3.8	19.9	21.2	5.0
Choice of endpoint categories (% of 1 + % of 4)	Mean	SD	Max	Min
ART	28.6	18.1	99.2	3.1
TRT	32.9	16.0	97.7	8.5

## Linear regression models

Table 6 shows the *F*-statistics of the winning models for each test, revealing that the ART model explains 64.2% of the variance and the TRT model explains 88.3% of the variance. We checked multicollinearity diagnostics (variance inflation factor [VIF]) and found that they were sufficiently low, with a maximum value of 1.698.

**Table 6:** Overview of the winning models for ART and TRT

Linear regression ANOVA	*F*	*df*	Sig.	*R*	*R*^2^	*F* Change	*df*2	Sig. *F* Change
d′ Author / Demographics	98.573	5	0.001	0.801	0.642	4.036	275	0.046
d′ Title / Demographics	328.046	3	0.001	0.883	0.780	5.376	277	0.021

The stepwise linear regression model for ART scores revealed significant effects for age, field of study, educational status, and writing habits (cf. Table 7). Recall that we used treatment coding for the categorical fixed effects. Accordingly, the notation of the factor variables (listed in the first column under “item” in Tables 7 and 8) gives the factor name to the left of the colon and the factor level that shows a significant effect vis-à-vis the reference level to the right of the colon. Performance on the ART improved (i.e., scores are higher) with increasing age of the participants. A degree in higher education (PhD) and personal writing habits (creative writing) also improved ART scores. We will not interpret the educational factor, because of the low sample size (*n* = 3) of the factor level ”PhD or equivalent” and because all three participants with the highest educational level (PhD) were enrolled in linguistics. Most importantly, we find that the field of study impacts ART scores. Students of comparative literature and German studies were significantly better than students of English studies. The other fields of study did not differ statistically from the reference level, English studies.

**Table 7:** Results of the winning stepwise linear regression model for the ART

Linear regression d′ Author / Demographics	Unstandardized coefficients		Standardized coefficients	*t*	Sig.
Item	B	Std. error	Beta		
Age	0.017	0.001	0.576	12.256	<0.001
Field of study: comparative literature	0.380	0.077	0.208	4.953	<0.001
Education level: PhD or equivalent	0.872	0.248	0.129	3.518	0.001
Creative writing: yes	0.114	0.057	0.094	2.015	0.045
Field of study: German studies	0.147	0.073	0.083	2.009	0.046

**Table 8:** Results of the winning stepwise linear regression model for the TRT

Linear regression d′ Title / Demographics	Unstandardized coefficients		Standardized coefficients	*t*	Sig.
Item	B	Std. Error	Beta		
Age	0.031	0.001	0.873	25.233	<0.001
Field of study: comparative literature	0.218	0.066	0.101	3.295	0.001
Gender: male	−0.135	0.058	−0.075	−2.319	0.021

The stepwise linear regression model for the TRT revealed significant effects for age, field of study, and gender (see Table 8). Performance on the TRT improved with the increasing age of the participants. Male participants had lower scores than female participants. Finally, students of comparative literature showed better test results compared with the reference level, English studies.

In summary, both ART and TRT were reliably influenced by the age of students and their field of study. There is a reliable distinction between students of comparative literature and English studies in both tests, and between students of German studies and English studies in the ART. As previously reported, test performance increases with increasing age. Gender influenced TRT but not ART scores. Variables such as the amount of time participants spent reading per week did not significantly influence the scores of either test.

### Item regression analysis

Regression analysis is a useful tool on the one hand to reduce the number of items for future development of the questionnaire with a view to analyzing d′. On the other hand, it allows checking the value of the fake author names and fake titles, as they show themselves as being less often chosen. With these aims in mind, we performed the regression analysis with a stepwise inclusion of items and the dependent variable d′ separately for authors and titles.

Recall that there are equal numbers of real and fake authors and equal numbers of real and fake titles (65 each). On average, real authors were correctly identified by 60.5% of the participants, while foils were correctly identified as being fake authors by 56.5% of the participants. The range in the case of real authors was 97.7% (for J.K. Rowling) to 30.0% (the Swiss popular science author Rolf Dobelli). The correct rejection of fake names ranged from 75.2% (Jürgen Burger) to 32.4% (Achim Rodenkamp), so every foil was selected at least once. Existing titles were selected with a significantly higher rate of correct choices: Real titles were recognized by 71.5% of the participants, which is better than the rejection of fake titles at 52.2% of the participants. Selection rates of real titles ranged from 99.3% (*Der* *kleine Prinz* “The little Prince,” *Das Parfüm* “The perfume”) to 35.3% (*Die* *Schnupftabaksdose. Stumpfsinn in Versen und Bildern* “The Snuff Box. Dullness in verse and pictures”). Rates for the correct rejection of fake titles ranged from 85.1% (*Die* *gejagten Erdnüsse* “The chased peanuts”) to 21.1% (*Unersättlich: Kapitalismus im 21. Jahrhundert* “Insatiable: Capitalism in the 21st century”).

Table 9 shows the *F*-statistics of the winning linear regression models for each test, revealing that the ART model in the item analysis explains 90.1% of the variance and the TRT model explains 95.5% of the variance.

**Table 9:** Overview of the winning models for ART and TRT

Linear regression ANOVA	*F*	*df*	Sig.	*R*	*R*^2^	*F* change	*df*2	Sig. *F* change
d′ Author / Items	67.812	29	<0.001	.949	0.901	3.986	217	0.047
d′ Title / Items	126.401	32	<0.001	.977	0.955	4.241	190	0.041

The winning model for authors (*R*^2^ = 0.901, *F* change = 3.986, ANOVA *p* < 0.001) includes 15 real and 14 fake authors.

Table 10 gives an overview including item difficulty (% correct) and the information on whether or not the author belongs to the group of canonical authors.

**Table 10:** Item difficulty (% correct responses for real or fake items) and information about canonicity for items in the ART

Linear regression d′ Author / Items			Unstandardized coefficients		Standardized coefficients		
Item	Real/ Foil	% Corr	B	Std. error	Beta	*t*	Sig.
Rolf Dobelli	R	30.0	−0.120	0.025	−0.481	−4.776	<0.001
Thomas Piketty	R	43.6	−0.083	0.025	−0.318	−3.336	0.001
Joachim Ringelnatz ^#^	R	61.4	−0.094	0.019	−0.316	−5.037	<0.001
Paulo Coelho	R	58.1	−0.092	0.019	−0.311	−4.858	<0.001
Alfred Döblin ^#^	R	63.0	−0.083	0.020	−0.269	−4.137	<0.001
Michel Houellebecq ^#^	R	42.6	−0.043	0.018	−0.160	−2.318	0.021
Axel Hacke	R	32.0	−0.039	0.019	−0.157	−1.996	0.047
Ken Follett	R	85.1	−0.060	0.024	−0.135	−2.494	0.013
Sebastian Fitzek	R	90.1	−0.060	0.022	−0.134	−2.677	0.008
Heinrich Böll^#^	R	83.8	−0.054	0.023	−0.131	−2.357	0.019
Antoine de Saint-Exupéry^#^	R	76.6	−0.048	0.019	−0.131	−2.512	0.013
Stephenie Meyer	R	83.2	−0.052	0.019	−0.129	−2.761	0.006
Stieg Larsson	R	85.1	−0.052	0.023	−0.125	−2.307	0.022
Alice Schwarzer	R	79.9	−0.035	0.016	−0.101	−2.209	0.028
Günter Grass ^#^	R	88.8	−0.041	0.023	−0.094	−1.771	0.078
Nils Hein	F	66.0	0.096	0.028	0.377	3.393	0.001
Josephine Wolf	F	49.8	0.095	0.023	0.344	4.058	<0.001
Antoine Bennett	F	46.5	0.085	0.022	0.307	3.911	<0.001
Frauke Cohrs	F	66.0	0.073	0.028	0.290	2.588	0.010
Victoria Bonneville	F	52.5	0.077	0.029	0.289	2.674	0.008
Carsten Caspari	F	51.8	0.072	0.021	0.275	3.370	0.001
Aiden Edwards	F	59.7	0.067	0.024	0.259	2.815	0.005
Monica Dietrich	F	52.5	0.063	0.025	0.234	2.485	0.014
Petra Fritsch	F	54.5	0.060	0.025	0.226	2.418	0.016
Vidar Sjögren	F	54.5	0.058	0.026	0.222	2.190	0.030
Isabelle Flores	F	39.9	0.062	0.025	0.219	2.458	0.015
Brigitte Novak	F	41.6	0.055	0.021	0.198	2.678	0.008
Harald Voß	F	53.5	0.052	0.025	0.196	2.115	0.036
Joaquim Herrera	F	46.9	0.052	0.024	0.187	2.134	0.034

^#^ canonical author

The winning model for titles (*R*^2^ = 0.955, *F* change = 4.241, ANOVA *p* < 0.041) includes 14 real and 18 fake titles.

Table 11 gives an overview including item difficulty (% correct).

**Table 11:** Item difficulty (% correct responses for real or fake items) and information about canonicity for items in the TRT

Linear regression d′ Title / Items			Unstandardized coefficients		Standardized coefficients		
Item	Real/ Foil	% Corr	B	Std. error	Beta	*t*	Sig.
Harry Potter und der Stein der Weisen	R	99.0	−0.346	0.069	−0.443	−4.981	<0.001
Das Kapital im 21. Jahrhundert	R	66.7	−0.088	0.018	−0.254	−4.973	<0.001
Verbrechen: Stories	R	37.3	−0.069	0.020	−0.230	−3.523	0.001
Regentonnenvariationen. Gedichte	R	38.0	−0.066	0.017	−0.227	−3.943	<0.001
Der kleine Prinz	R	99.3	−0.155	0.048	−0.210	−3.232	0.001
Der Flammenträger	R	54.1	−0.060	0.021	−0.189	−2.899	0.004
Die Blechtrommel	R	84.5	−0.079	0.018	−0.159	−4.306	<0.001
Unterwerfung	R	74.6	−0.058	0.017	−0.148	−3.395	0.001
Altes Land	R	73.3	−0.046	0.018	−0.127	−2.602	0.010
Sakrileg	R	92.4	−0.073	0.022	−0.125	−3.290	0.001
Bob. der Streuner	R	63.4	−0.045	0.014	−0.125	−3.243	0.001
Der Vorleser	R	96.7	−0.079	0.034	−0.115	−2.287	0.023
Erbarmen	R	62.7	−0.033	0.017	−0.096	−1.978	0.049
Der Hundertjährige, der aus dem Fenster stieg und verschwand	R	90.8	−0.046	0.023	−0.078	−1.976	0.050
Croissants zum Frühstück: Roman	F	43.2	0.108	0.018	0.334	5.925	<0.001
Blumen im Schatten	F	43.9	0.101	0.021	0.314	4.843	<0.001
Mit den Augen Goethes – Reise nach Rom	F	37.6	0.081	0.020	0.237	3.970	<0.001
Traumatisch: Vergiftete Kindheit und ihre Folgen	F	55.8	0.067	0.020	0.225	3.332	0.001
Wackelohr	F	67.7	0.062	0.023	0.217	2.736	0.007
Das Licht am Bodensee	F	55.4	0.063	0.023	0.205	2.705	0.007
Harrys Alter Ego	F	66.0	0.056	0.018	0.196	3.128	0.002
„Alles Banane“ – Wege zu einer positiven Einstellung	F	49.5	0.060	0.019	0.194	3.226	0.001
Regenwolken über Rom	F	50.8	0.060	0.023	0.192	2.619	0.010
Regentropfen	F	61.1	0.051	0.020	0.171	2.602	0.010
Die Rückseite des Glücks	F	36.3	0.056	0.019	0.168	2.934	0.004
Zurück nach Panama	F	23.8	0.062	0.019	0.158	3.330	0.001
Das Funkeln des Wassers	F	39.6	0.052	0.020	0.155	2.588	0.010
Untötbar	F	61.1	0.041	0.020	0.142	2.059	0.041
Über den Dächern von Berlin-Kreuzberg	F	34.7	0.046	0.020	0.136	2.358	0.019
Zeugma und andere Verwirrungen	F	68.0	0.035	0.020	0.120	1.735	0.084
Durch die Felder und zurück	F	48.8	0.038	0.020	0.118	1.879	0.062
schwarzgrau: Gedichte	F	47.9	0.036	0.017	0.116	2.104	0.037

There is also a significantly higher recognition rate for “canonical” authors. The mean recognition rate for “canonical” authors is 70.0%, while 56.6% of noncanonical real authors are correctly recognized (*t*-test for independent samples *t* = 2.791, *df* = 63, *p* = 0.007). The recognition of “canonical” titles in the TRT could not be analyzed because there is too great a variety of titles written by “canonical” authors in the different school curricula.

## Discussion

Our results can be summarized as follows: ART and TRT performance is partly predicted by the field of study that the participants are enrolled in. From the list of demographic predictors, those of reading and writing habits, age, educational level, and creative writing predict ART and TRT performance. Gender predicts TRT performance.

The current study investigated performance in the ART and TRT with regard to three related research questions.

First, we examined whether, for a student population, the field of study influences test results, by contrasting academic subjects from the humanities and sciences (German studies, comparative literature, English studies, linguistics, psychology, and medicine). Our results show that academic background makes a difference in some instances: students of comparative literature performed best overall. The differentiated impact of academic background suggests that ART/TRT scores do not simply reflect print exposure in quantitative terms, i.e., the number of fiction texts that students are required to read for their studies. Rather, it seems that a clear focus on fiction in a study program of the humanities supports successful author and title recognition: Students of comparative literature are highly focused on reading works of a more varied range of (international) authors (i.e., they read works in three different languages as per the study requirements of the universities where we obtained our samples). As a result, they are probably more aware of the works of authors publishing in German and other languages in general, which shows up as better test performance in the ART/TRT. As McCarron and Kuperman (2021: 2227) put it: “the assumption is that a greater amount of reading leads to a greater awareness of the existing literature and reading sources, which translates into higher recognition scores.” This is supported by the finding that students of German studies also score higher than students of English studies in the ART. Their study program includes more material from the list of authors used in our German version of the ART than the study program in English studies. Overall, this can be seen as an instance of the Matthew effect, which suggests that those who possess a particular skill will also develop it further (Cunningham & Stanovich, 1990; Kempe et al., 2011; Protopapas et al., 2016).

Second, we hypothesized that recognition may vary depending on whether content (mnemonically associated with the title) or meta-literary information (authorship) is used as a cue to retrieve the information from memory. Here, we find that TRT scores are generally higher than ART scores, although the relative gradations between the fields of study are the same. Title recognition is easier for all participants in our study, and students of comparative literature show an additional advantage. This shows that the focus of a study program on fiction or nonfiction genres does not interact with how information is retrieved from memory to master the test. This may suggest that current practices of media consumption, which may put more emphasis on the book/text than the author, make successful performance in the TRT easier. Of course, this predicts that a different pattern should be obtained with a group of participants who engage less in convergent media practices (Jenkins, 2006).

Third, we included a questionnaire on demographics and reading and writing habits in order to use self-report measures as predictors for ART and TRT scores. We replicate the effect of age with our sample of university students (see Grolig et al., 2020 for ART): the older the participants are, the better their test scores. Our results disconfirm the hypothesis that print exposure reflects the mere “amount of text” read, as this would have predicted that the time someone spends reading should correlate positively with test performance. Finally, we find that a higher educational level and individual engagement in creative writing predicts higher test scores. We do not interpret the educational factor, because in our sample participants with the highest educational level all held a PhD in linguistics (*n* = 3). Thus, this finding suggests further replication in future research. Personal engagement in creative writing, by contrast, seems to be a reliable predictor of performance in both tests. Participants who write themselves recognize more authors and titles. This was an unpredicted finding which hints in the direction of general motivation: being involved in creative writing suggests greater interest in reading and writing generally and can lead to a higher level of literacy. Here, future research might yield further interesting results.

Finally, we used a 2afc method with a four-point scale which allowed participants to also indicate their rating confidence (see Table 5 in Results). This method is not a standard procedure in ART, TRT, or MRT. We decided to apply this alternative method in order to force a decision, evade problems in response bias, and gain a regression analysis identifying the most suitable authors for the creation of a new shorter ART/TRT. One of the differences between the traditional circling/checking of authors and the 2afc method is that participants need to make a differentiated appraisal of each name in the latter case instead of only looking for known items: this is indicated by the response confidence in whether an item is “certainly” or “presumably” recognized as a real or fake author/title. It becomes evident that for knowledge retrieval, i.e. the correct identification of real authors, there is the greatest confidence (17.5%), and less confident positive answers amount to 12.8%. Compared with the confidence in identifying foils (“certainly” 3.2% and “presumably” 18.5%), the difference aptly reflects the fact that if you know an author you can have certainty, while knowing that someone is *not* an author is basically impossible. Thus, participants feel much less confident in their choice. The great advantage of the method chosen for the present is getting a clearer picture of the quality of the answers in regard to certainty. However, as we did not compare the two response methods in our study, we do not have conclusive insights into the different processing of the decision-making. Nevertheless, the confidence rating is included in our item regression analysis and thereby contributes to the evaluation of item strength when it predicts test performance.

## Limitations

One limitation of this study is the large number of questionnaires which had to be excluded from the study: out of 410 completed questionnaires, 107 had to be excluded, with the majority (50) of them because of aberrant behavior of the students in filling in the questionnaires. This problem might have arisen because students could not be sufficiently motivated to participate meaningfully in the test. This might have been because of the time required to fill in all the questionnaire items. Certainly, it remains an open question to what extent individual motivation generally affects test performance in the ART/TRT, and future research may include assessments to further scrutinize motivational profiles. We have suggested that using regression analysis with the individual items as predictors may allow for systematic selection of authors or titles for future tests, which will then present a shorter list of items. In addition, with a view to the speed of performing the test, the traditional checking/circling is most probably more time-efficient than the four-point scale we used.

A general limitation observable in the ART/TRT is the selection of authors/titles and the question of whether such a list is a representative sample of the entire range of authors/titles individuals are familiar with: there are genre-specific (e.g., crime novels, fantasy) intense readerships who have extremely high levels of print exposure but will not find their genres represented in the typical ART/TRT list based on sales figures. A possible solution to this conundrum could be the addition of an active recollection task: “Write down as many author names/titles as you can in 1 minute.”

Another limitation concerns the measurement of the quantitative amount of reading participants engage in during a week. We did not find any significant influence of the average weekly reading time on ART/TRT performance; however, this may be a consequence of the wording of the closed-ended questions in our questionnaire (i.e., how are genres defined by participants, how do they determine reading time?).

## Future directions

In summary, we have argued that it is far from trivial to operationalize print exposure. It is, evidently, not the mere “amount of text” read (Acheson et al., 2008, p. 278) or the number of hours spent reading, but the composition of what someone reads and possibly also the influence of media convergence, i.e., the diversification of information channels through which cultural knowledge is gathered (Jenkins, 2006; Sparviero et al., 2017).

The overall pattern in our data suggests that ART and TRT should be developed and administered with an eye to the demographic and habitual characteristics of the group of participants to be sampled. Author names and titles should be selected based on the sample’s actual reading behavior. That is, if the sample indicates (via demographic or self-report questionnaires) that they read fiction in particular, the genre should be reflected in the test items. The same principle applies to other genres such as nonfiction.

Additionally, we determined that, contrary to what Stanovich and West (1989) proposed, canonical authors should be part of the ART test. In our study, about a third of the canonical (fiction) authors proved to be significant predictors of ART scores. Thus, along with the strengthening of specific genres in the test materials, canonicity per genre should also be considered. Hence, ART/TRT test items should be updated on a regular basis, especially regarding online reading which may contain information on authors or titles that are currently the subject of public attention.

In terms of age, it seems that participant samples for which test estimates are derived and/or compared with one another should be as homogeneous as possible to allow for meaningful comparison. For adult groups of participants, age has to be considered a robust factor even for seemingly homogeneous groups.

In general, independently of the language in which ART/TRT are employed, resulting test scores are low. Stanovich and West (1989) have a “mean performance on the MRT (23.8, *SD* = 6.3)” and “on the ART (9.3, *SD* = 5.7)” (Stanovich & West, 1989, 410), Acheson et al. (2008) report mean selection rates of 36% (ART) and 37% (MRT), Moore and Gordon (2015) found a mean recognition rate for the ART of 23.8%, and Grolig and colleagues (Grolig et al., 2020) report a 24.36% recognition rate in their ART. Our own results are no exception to this: our participants recognized with certainty 17.5% of real authors and with less certainty 12.8%, which combined to an overall recognition of 30.3%. This correspondence is intriguing in light of the different scales that were used in our study and in previous ones (see above in the Discussion on scale formats). Our mean d′ values were between 0.319 and 0.830 for the ART and 0.556 and 0.894 for the TRT. So, participants in our study showed rather modest recognition rates overall (recall that d′ = 0 indicates guessing). This may indicate that it is important to design the ART/TRT specifically for the participant sample under investigation (see above).

Finally, we conclude that a combination of different measures may be better suited to operationalize print exposure. The combination of ART and TRT seems to better capture different memorization strategies associated with either author names or titles. The combination of ART/TRT and demographic and habitual self-report measures enhances the construct validity of ART/TRT (or, vice versa, reduces the specification error), allowing for item selection that is more sensitive to reading-based knowledge.

## Data Availability

The raw data used for analysis and an example of the questionnaire as given to the participants are publicly available at https://osf.io/s6j2e/?view_only=7e8f0f874570449eb7ce0865f6d9e38b.
